# Does Tyrosyl DNA Phosphodiesterase-2 Play a Role in Hepatitis B Virus Genome Repair?

**DOI:** 10.1371/journal.pone.0128401

**Published:** 2015-06-16

**Authors:** Xiuji Cui, Rebecca McAllister, Rajeev Boregowda, Ji A. Sohn, Felipe Cortes Ledesma, Keith W. Caldecott, Christoph Seeger, Jianming Hu

**Affiliations:** 1 Department of Microbiology and Immunology, Hershey, The Pennsylvania State University, College of Medicine, Hershey, Pennsylvania, United States of America; 2 Fox Chase Cancer Center, Philadelphia, Pennsylvania, United States of America; 3 Centro Andaluz de Biología Molecular y Medicina Regenerativa (CABIMER)—CSIC, Av. Américo Vespucio s/n, 41092 Sevilla, Spain; 4 Genome Damage and Stability Centre, University of Sussex, Science Park Road, Falmer, Brighton, Sussex BN1 9RQ, United Kingdom; Academia Sinica, TAIWAN

## Abstract

Hepatitis B virus (HBV) replication and persistence are sustained by a nuclear episome, the covalently closed circular (CCC) DNA, which serves as the transcriptional template for all viral RNAs. CCC DNA is converted from a relaxed circular (RC) DNA in the virion early during infection as well as from RC DNA in intracellular progeny nucleocapsids via an intracellular amplification pathway. Current antiviral therapies suppress viral replication but cannot eliminate CCC DNA. Thus, persistence of CCC DNA remains an obstacle toward curing chronic HBV infection. Unfortunately, very little is known about how CCC DNA is formed. CCC DNA formation requires removal of the virally encoded reverse transcriptase (RT) protein from the 5’ end of the minus strand of RC DNA. Tyrosyl DNA phosphodiesterase-2 (Tdp2) was recently identified as the enzyme responsible for cleavage of tyrosyl-5’ DNA linkages formed between topoisomerase II and cellular DNA. Because the RT-DNA linkage is also a 5’ DNA-phosphotyrosyl bond, it has been hypothesized that Tdp2 might be one of several elusive host factors required for CCC DNA formation. Therefore, we examined the role of Tdp2 in RC DNA deproteination and CCC DNA formation. We demonstrated Tdp2 can cleave the tyrosyl-minus strand DNA linkage using authentic HBV RC DNA isolated from nucleocapsids and using RT covalently linked to short minus strand DNA produced *in vitro*. On the other hand, our results showed that Tdp2 gene knockout did not block CCC DNA formation during HBV infection of permissive human hepatoma cells and did not prevent intracellular amplification of duck hepatitis B virus CCC DNA. These results indicate that although Tdp2 can remove the RT covalently linked to the 5’ end of the HBV minus strand DNA *in vitro*, this protein might not be required for CCC DNA formation *in vivo*.

## Introduction

An estimated 350 million persons worldwide are chronically infected with hepatitis B virus (HBV), 25% of whom will die from severe liver diseases including cirrhosis and hepatocellular carcinoma [1]. HBV belongs to the *Hepadnaviridae* family of viruses, which include the duck hepatitis virus (DHBV). All hepadnaviruses contain a small (ca. 3.2 kb) relaxed circular (RC), partially double-stranded DNA genome that replicates via reverse transcription through an RNA intermediate called pregenomic RNA (pgRNA) [2–4]. Upon infection of hepatocytes, the genomic RC DNA is transported to the nucleus and converted (repaired) to an episomal covalently closed circular (CCC) DNA [5] that serves as the template for transcription of all viral RNAs. Unlike conventional retroviral reverse transcription, hepadnaviruses initiate minus or (-) strand DNA synthesis using a tyrosine (Y) residue within a unique terminal protein (TP) domain of the virally encoded reverse transcriptase (RT) itself as a primer (protein priming), a process that results in the covalent linkage of RT to the 5’ end of the (-) strand of RC DNA [6–9]. Upon completion of reverse transcription, mature RC DNA-containing nucleocapsids (NCs) are enveloped by viral surface proteins and secreted as virions or, alternatively, RC DNA is recycled back to the nucleus to amplify the pool of CCC DNA [5, 10, 11].

HBV infections are able to persist due to the formation and persistence of approximately 10–50 copies of CCC DNA within the nuclei of each infected hepatocyte [5, 10, 12]. Unfortunately, current therapies that inhibit HBV RT polymerase activity have no direct effect on CCC DNA formation. CCC DNA can persist even after years of antiviral therapy and is responsible for the rapid rebound of viral replication after treatment cessation [4, 13]. Thus, elimination of CCC DNA is of paramount importance for a cure for chronic HBV infection.

CCC DNA formation requires removal of RT from the 5’ end of the (-) strand DNA and a capped, 18 nucleotide-long RNA from the 5’ end of the plus strand DNA. In addition, one of the 9 nucleotide-long terminally redundant segments on the (-) strand DNA, termed r, has to be removed before ligation of the 5’ and 3’ ends of the two DNA strands can occur. In the case of the plus strand DNA, which is typically incomplete in cytoplasmic core particles and virions, extension of the DNA strand must also occur before the ends can be joined. The mechanism and pathway of RC to CCC DNA conversion remain unknown. Recently, a form of RC DNA free of RT (thus called protein-free [PF] or deproteinated [DP] DNA) was identified in HBV-transfected hepatoma cell lines [11, 14]. The same putative PF-RC form may also have been detected in HBV transgenic mouse liver [15], in HBV infected hepatoma cells [16], and in DHBV infected primary duck hepatocytes under certain conditions [17]. It is currently unknown how this PF-RC DNA is generated. Further studies on the mechanism of its generation could provide insights into CCC DNA formation as RC DNA deproteination is a prerequisite for RC to CCC DNA conversion.

Viral and host factors involved in CCC DNA formation also remain to be defined. The large surface (LS) protein of DHBV suppresses CCC DNA amplification via a negative feedback mechanism [17, 18]. Thus, disruption of DHBV LS protein expression by deletion, or specific substitution mutations, increases CCC DNA levels more than 10 fold [17, 18]. Recently, a lack of HBV envelope protein expression was also shown to lead to an increase, though more modest than in DHBV, in CCC DNA formation in HBV-replicating cell lines [11, 14, 19], supporting a general role of hepadnaviral envelope protein in the regulation of CCC DNA formation.

Specific host factors directly involved in CCC DNA synthesis remain to be identified. A potential host factor involved in CCC DNA conversion was characterized recently while studying cellular DNA damage repair [20, 21]. Covalent protein-DNA adducts are common cellular lesions that must be continuously repaired for cell growth and survival. Topoisomerase (Topo) I and II-DNA adducts are examples of such lesions [22, 23]. While tyrosyl DNA phosphodiesterase-1 (Tdp1) breaks tyrosyl-3’ DNA linkages characteristic of Topo I-DNA adducts [23], the recently identified tyrosyl DNA phosphodiesterase-2 (Tdp2), a multifunction protein, previously known as TNF receptor-associated factor (TRAF) and TNF receptor-associated protein (TTRAP), efficiently cleaves tyrosyl-5’ DNA linkages present in Topo II-DNA adducts [20, 21]. Structurally, the linkage between RT and the 5’ end of the (-) strand of RC DNA is identical to the 5’ DNA-phosphotyrosyl bonds of Topo II-DNA adducts. Indeed, we and others have recently reported that purified human Tdp2 can cleave the RT-(-) DNA linkage the same way as it does the Topo II-DNA linkage [9, 24, 25]. Furthermore, some evidence has been presented very recently suggesting that human Tdp2 may facilitate DHBV CCC DNA formation in human cells via the intracellular amplification pathway [25]. We have carried out further biochemical analysis of the cleavage of the RT-DNA linkage by Tdp2 and determined its role in HBV CCC DNA formation during both viral infection and intracellular CCC DNA amplification using cells with CRISPR/Cas9 mediated genetic knockout of Tdp2.

## Results

### Tdp2 cleaved the 5’-phosphotyrosyl linkage between the RT protein and the 5’ end of the (-) strand of RC DNA

**Fig 1:**
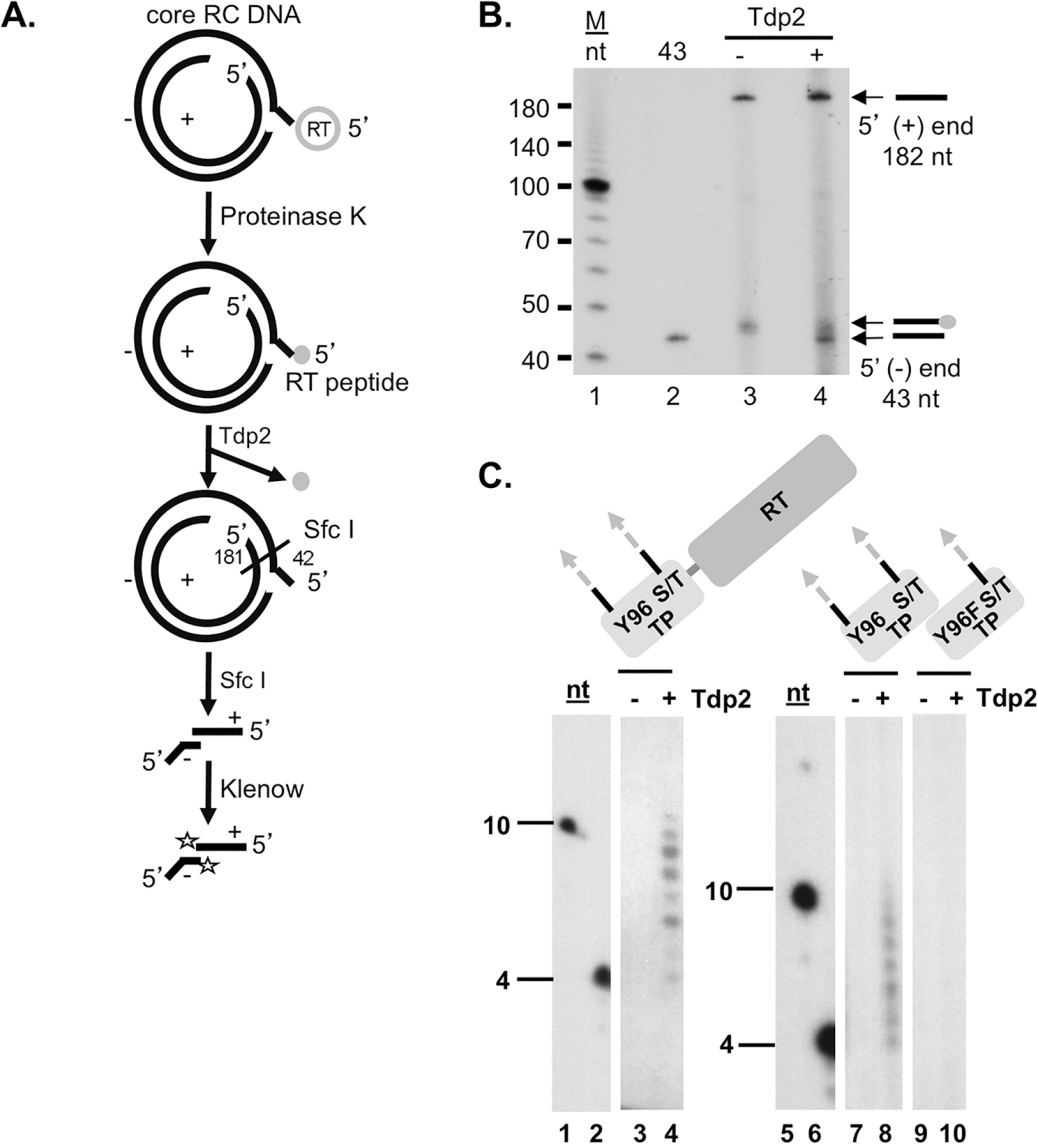
Tdp2 cleavage of the covalent linkage between the RT protein and the 5’ end of the viral minus strand DNA. (A) and (B). Tdp2 removal of proteinase K-derived RT peptide from the 5’ end of core RC DNA. (A) Diagram of the Tdp2 cleavage reaction and detection of the cleavage product derived from the HBV RC DNA. HBV DNA extracted from cytoplasmic nucleocapsids (core) with proteinase K digestion was treated with Tdp2, followed by Sfc I digestion and labeling with [α-^32^P]-TTP using Klenow fill-in reaction. Proteinase K generated RT peptide is indicated by a small shaded circle. The restriction endonuclease Sfc I cuts near the 5’ end of the (-) strand DNA, within the cohesive overlap of RC DNA, generating fragments containing short 5’ (-) and (+) DNAs 42 and 181 nt (the RNA primer being removed by prior RNase digestion) in length, respectively, as indicated. A single radiolabeled TMP added by Klenow to the 3’-recessed ends of the Sfc I-generated core RC DNA fragments are indicated by the stars. (B) Klenow-labeled products were resolved by urea-polyacrylamide gel electrophoresis (PAGE) and visualized by autoradiography. The 5’ ends of the (+) and (-) strand of core RC DNA are indicated by arrows as are the 5’ end of the (-) strand with or without the residual RT peptide. The lane labeled as “43” contained a DNA oligonucleotide having the same sequence as the first 43 nt of the HBV (-) strand DNA and labeled at the 5’ end by using T4 ploynucleotide kinase and [γ-^32^P]-ATP. (C)**.** Tdp2-mediated cleavage of DHBV RT-DNA linkages generated *in vitro*. MiniRT2 protein priming reactions carried out in the presence of Mn^2+^, [α-^32^P]-dGTP and unlabeled dATP, dCTP, dTTP, were mock treated (-) or treated (+) with Tdp2 (lanes 3 and 4). TP, either WT (lanes 7 and 8) or Y96F mutant (lanes 9 and 10), was mixed with the polymerase domain for *trans*-complementation priming reaction in the presence of Mn^2+^, [α-^32^P]-dGTP and unlabeled dATP, dCTP, dTTP. The reactions were mock treated (-) or treated (+) with Tdp2 (lanes 7–10). Reaction products from mock treated (-) or Tdp2-treated (+) protein priming reactions were resolved by urea-PAGE and detected by autoradiography. Lanes 1, 2, 5, and 6 show the 10 and 4 nt DNA marker. Domain diagrams of MiniRT2, TP and its mutant derivative Y96F-TP are depicted above each panel with the primer residue Y96 or F substitution indicated. The DNA oligomers covalently attached to the MiniRT2 protein or the TP domain are highlighted by the shaded and dotted arrows extending from the protein. Cryptic serine and threonine (S/T) residues that can serve as priming sites under Mn^2+^ conditions, as well as the authentic Y96 site, are highlighted.

A recent study showed that pretreatment of the HBV and DHBV RC DNA–RT complex released from NCs with Tdp2 could make the 5’ end of the (-) strand susceptible to 5’ exonuclease digestion, suggesting that Tdp2 could release the RT protein from RC DNA [25]. To directly monitor the cleavage of the phosphotyrosyl linkage by Tdp2, we extracted HBV DNA in cytoplasmic core particles with proteinase K, which leaves a peptide derived from the RT attached to the 5’ end of the (-) strand (Fig 1A). Incubation with Tdp2 should cleave the tyrosyl-DNA bond and yield protein free minus strands. To assess this possibility experimentally, proteinase K-digested HBV RC DNA was treated with Tdp2, digested with the restriction endonuclease Sfc I and radioactively labeled with a Klenow fill-in reaction in the presence of [α-^32^P]-TTP. The DNA products of this reaction were resolved by urea- PAGE, and visualized by autoradiography. The autoradiograph showed the two expected DNA products from the Sfc I digestion and Klenow fill-in reaction of 43 and 182 nt derived from the minus and plus strand DNA, respectively (Fig 1B, lanes 3 and 4). Moreover, the results showed that Tdp2 hydrolyzed the phosphodiester bond between the RT-derived peptide and the 5’ end of the minus strand DNA because the mobility of the RT peptide-43mer shifted to a slightly faster species in the presence of Tdp2, which co-migrated with a DNA oligomer representing the 5’ most 43 nt of the minus strand DNA (Fig 1B, lanes 2 and 3). Thus, the RT peptide-linked RC DNA was a substrate for Tdp2 cleavage *in vitro*.

To investigate whether Tdp2 could cleave the tyrosyl-phosphodiester bond between DNA and enzymatically active RT, we used an *in vitro* protein priming reaction to produce RT proteins covalently attached to a short DNA oligomer several nt long via the phosphotyrosyl bond [26].To facilitate the priming reaction, we used a truncated RT termed MiniRT2 that is active when purified from bacteria [27]. In addition, we used a two-component system for the DNA priming reaction, where the TP and polymerase domains of the RT are expressed separately [26, 28]. The products of the protein priming reactions were then incubated with Tdp2 to release the radiolabeled DNA oligomers, which were then detected by urea-PAGE [9, 24]. As shown in Fig 1C, Tdp2 was able to release DNA oligomers from either MiniRT2 or TP priming products (lanes 4 and 8). To verify the specificity of Tdp2 for tyrosine-DNA linkages, we used a Y96F TP mutant that cannot form the phosphotyrosyl DNA linkage but allows formation of the non-physiological phosphoseryl and phosphothreonyl DNA linkages [26]. As expected, these linkages were not a substrate for Tdp2 cleavage (Fig 1C, lane 10).

Taken together, these results demonstrated that Tdp2 could cleave the 5’-phosphotyrosyl linkage between RT and the 5’ end of (-) strand DNA. The cleavage was not affected substantially by the size of the RT attached to the DNA or the size of the DNA attached to the protein. Finally, as expected Tdp2 cleavage was specific for tyrosine phosphodiester bonds, as observed with its natural function during the release of Topo II from DNA.

### Tdp2 knockout cells were permissive to HBV infection

**Fig 2:**
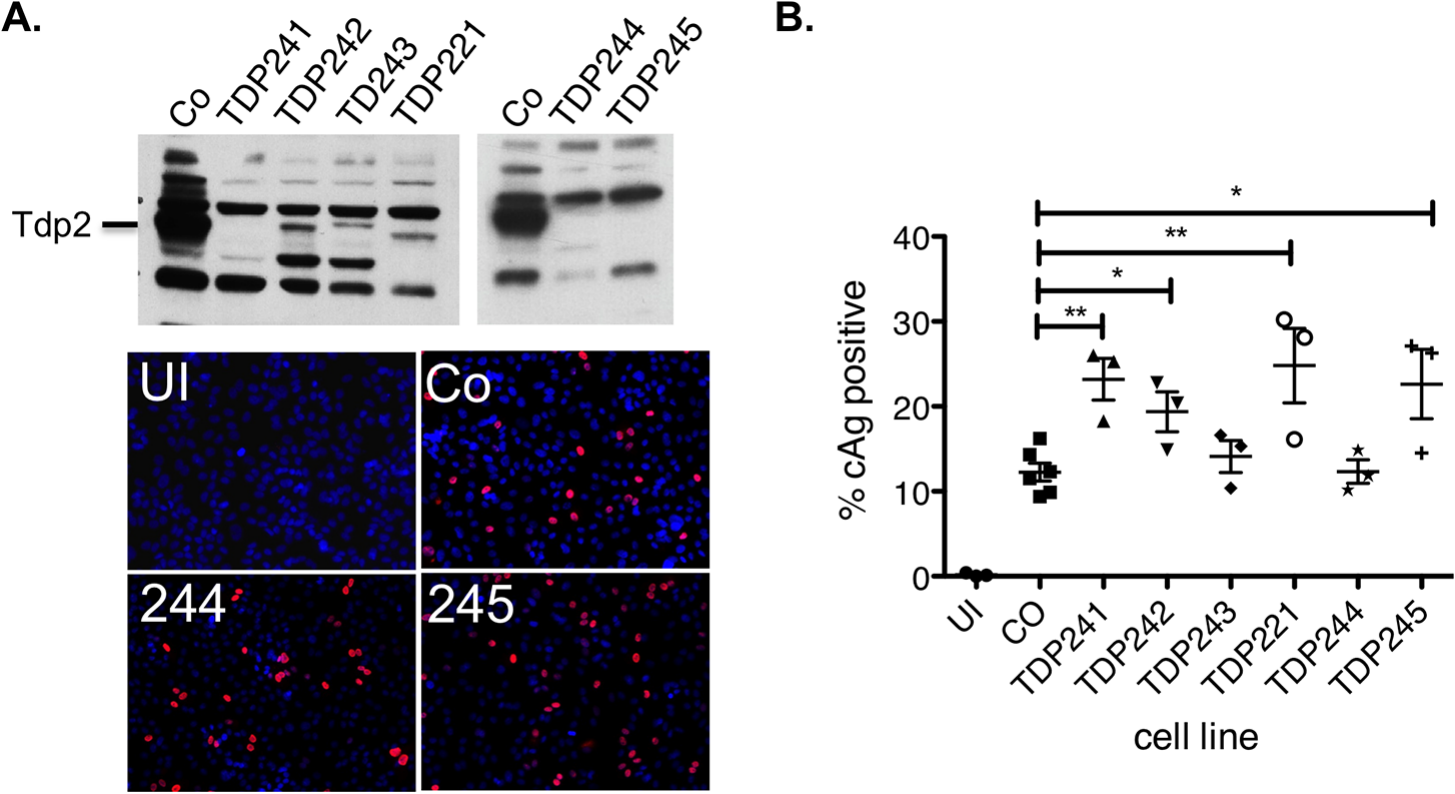
HBV infection in HepG2-NTCP Tdp2 knockout cells. HepG2-NTCP-Cas9 cells (control or Co) and six independent Tdp2-deficient clones (Tdp241, -242, -243, -221, -244 and -245) were infected with HBV. Western blots were developed with a rabbit anti-Tdp2 antibody (Bethyl Laboratories) to detect Tdp2 (A, top). Infected cells were visualized by immunofluorescence staining with an anti-HBc antibody (C1-5) eight days after HBV infection and selected images are shown (A, bottom; Co, 244, 245). Uninfected control cells were included as a negative control for the immunostaining (UI). (B) Quantitative analysis of infection efficiency as determined by HBc positive cells. *, p ≤ 0.05. **, p ≤ 0.01. Unpaired, two-tailed t-tests (Prism 5) were used for statistical analyses.

So far, our *in vitro* studies suggested the possibility that Tdp2 could play a role in the conversion of core RC to CCC DNA by mediating the removal of RT from the 5’ end of the (-) strand of RC DNA. To examine whether CCC DNA formation was dependent on Tdp2 activity in cell lines supporting HBV infection and DNA replication, we used a CRISPR/Cas9-based approach to mutate Tdp2 alleles in HepG2 cells to produce several knockout cell lines. Tdp2 knockout was confirmed by western blot analysis (Fig 2), DNA sequence analysis of the mutated alleles and cell growth assays in the presence of etoposide, a Topo II poison that induces covalent Topo II-DNA adducts, which require Tdp2 for repair (data not shown; [23]). To permit HBV infection, these cell lines also express the HBV receptor sodium taurocholate cotransporting polypeptide (NTCP) [29, 30]. As shown in Fig 2, cell lines TDP242 and TDP221 still expressed low levels of TDP2, possibly resulting from frame-shift mutations that could be partially reversed during translation. In the other four cell lines Tdp2 could not be detected. All six cell lines exhibited an approximately four-fold enhanced sensitivity to cell growth inhibition by etoposide (data not shown). All six cell lines remained susceptible to HBV infection as determined with an immunofluorescence assay using a HBV core protein (HBc)-specific antibody (Fig 2). Detection of HBc staining in this infection system is known to be dependent on functional CCC DNA formed from the incoming virion RC DNA [30]. The efficiency of HBV infection varied among cell clones and might have been even higher in the Tdp2 knockout cells compared with the control cells (Fig 2). However, the differences were small and might have been the result of random variations among different cell clones. Nevertheless, these results demonstrated clearly that Tdp2 is dispensable for HBV infections and hence, CCC DNA formation.

### Knockdown of Tdp2 was associated with a modest increase in HBV CCC DNA formation

**Fig 3:**
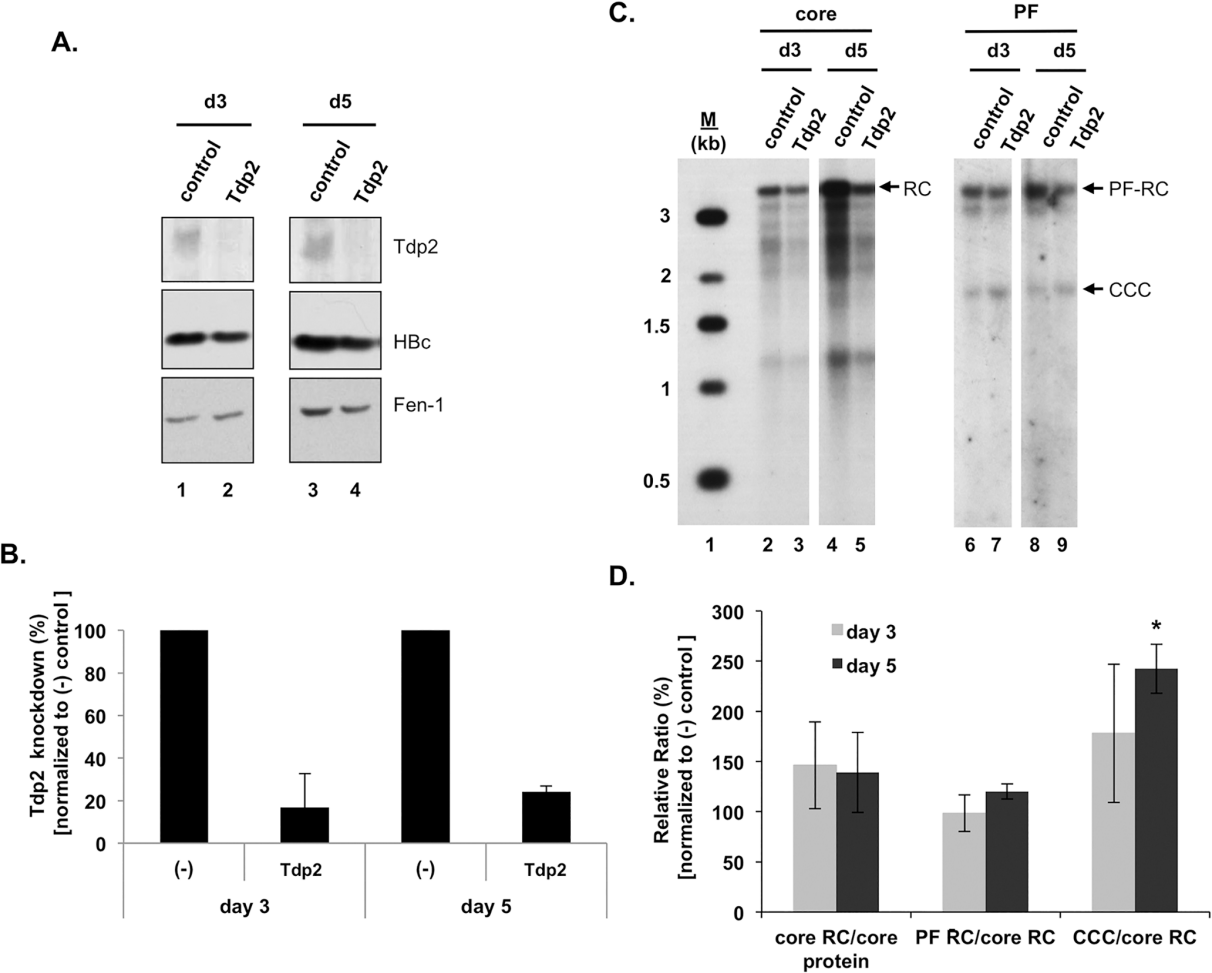
Knockdown of Tdp2 was associated with an increase in HBV CCC DNA. HBV replication was induced in HepAD38 cells by removal of tetracycline from the culture medium. Cells were transfected 4 days after tetracycline removal with control (Non-target) or Tdp2-specific siRNA. Viral DNA levels were measured 3 and 5 days post-siRNA transfection. (A) Tdp2 and HBV core protein levels were measured by western blot analysis. Fen-1 was used as a loading control. (B) Percent Tdp2 knockdown at day 3 and 5 post-siRNA transfection relative to control siRNA knockdown (set to 100%). (C) HBV DNAs were extracted from the cytoplasmic nucleocapsid (core) with protease digestion or the Hirt supernatant without protease digestion (PF) and analyzed by Southern blotting. (D) Quantitative analysis of HBV DNA replication. The indicated viral DNA ratios following Tdp2 knockdown were normalized to the corresponding DNA ratios after control (Non-target) treatment (set to 100%) and are reported as the mean relative ratios ± standard error of the mean of three independent experiments. The asterisk indicates a significant difference in Tdp2 knockdown CCC/core RC compared to control conditions at day 5 post-siRNA transfection (P < 0.05; unpaired, two-tailed t-test). CCC, CCC DNA; PF-RC, PF-RC DNA; RC, RC DNA.

To directly determine the effect of Tdp2 on HBV CCC DNA levels and specifically, the effect on the intracellular amplification pathway of CCC DNA formation, we employed the HepAD38 cells, These cells replicate HBV to high levels in an inducible manner and produce levels of HBV CCC DNA readily detectable by Southern blot analysis exclusively via intracellular amplification, as they are not susceptible to HBV infection [11, 31]. The cells were transfected 4 days after HBV induction with control (Non-target) or Tdp2-specific siRNA to reduce Tdp2 expression during CCC DNA formation and viral DNA levels were measured 3 and 5 days later. Fig 3A and 3B show that Tdp2 protein levels were markedly reduced (ca. 70–80%) following RNAi-mediated inhibition. We noticed that Tdp2 knockdown (or knockout, see below) appeared to modestly decrease HBV (Fig 3A) and DHBV (see below) core protein expression, which resulted in corresponding decreases in viral core DNA levels. The reason for this reduction in core DNA is not known, but might be explained by changes in the cellular environment caused by inhibition of additional activities attributed to Tdp2 in signal transduction [23]. To control for the pleiotropic effects of Tdp2 on the viral life cycle preceding CCC DNA formation, the effects of Tdp2 on CCC DNA levels were normalized to core RC DNA levels.

Consistent with the Tdp2 knockout results above, HBV CCC DNA remained readily detectable in the Tdp2 knockdown cells (Fig 3C). Furthermore, quantification of HBV CCC DNA levels showed that Tdp2 knockdown was associated with a modest increase of HBV CCC DNA when normalized to core RC DNA (Fig 3D, day 5). Viral core DNA levels were not affected when normalized to core protein levels (Fig 3D). Furthermore, HBV PF-RC DNA, which accumulates in cultured hepatoma or non-hepatoma cells to levels higher than CCC DNA (Fig 3C) [11], was not significantly affected by the reduction in Tdp2 either (Fig 3D). Together with the *in vitro* findings, these data suggest that Tdp2-mediated deproteination of RC DNA could suppress, rather than facilitate, HBV CCC DNA formation.

### Overexpression of Tdp2 was associated with a modest decrease in HBV CCC DNA formation

**Fig 4:**
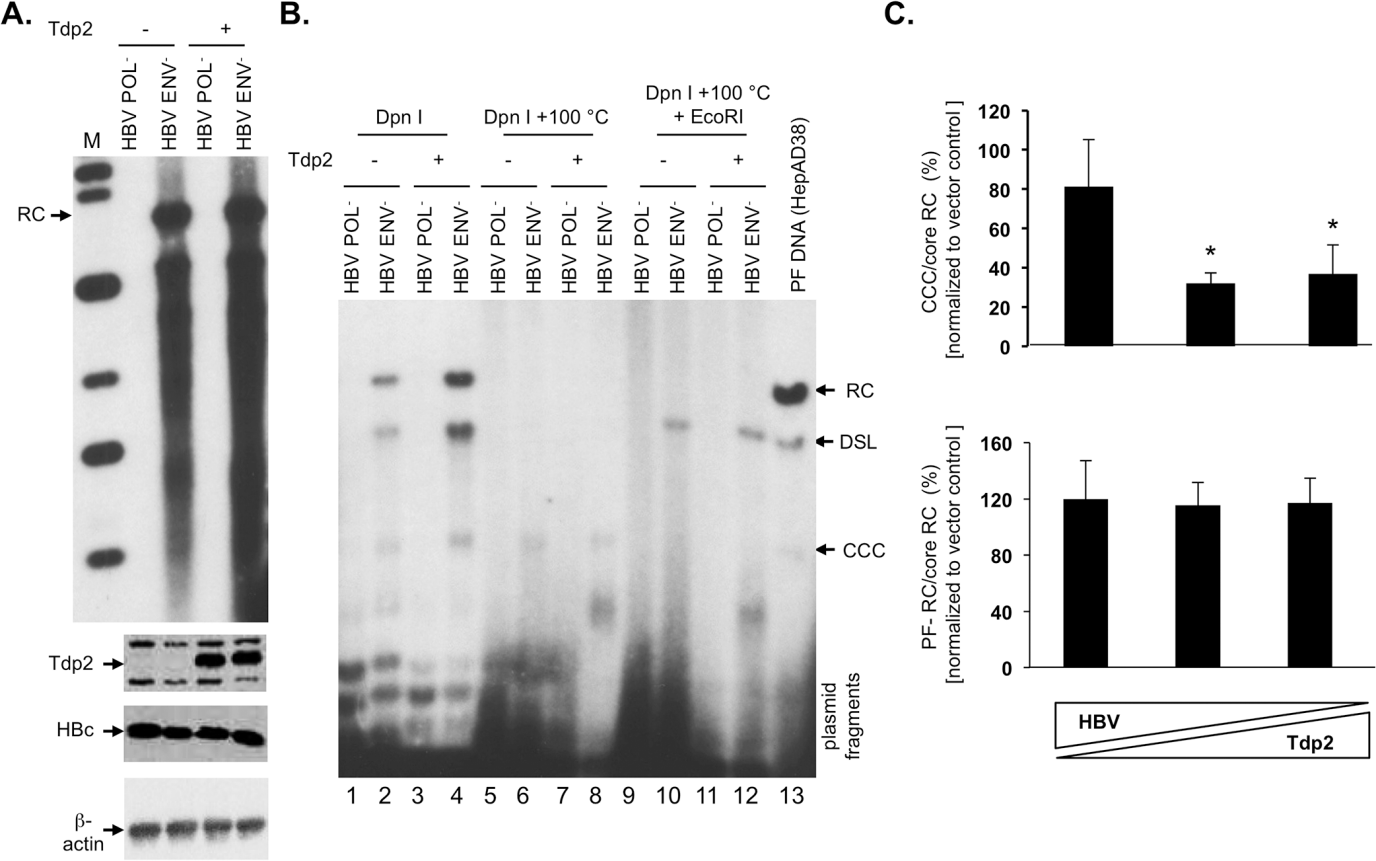
Overexpression of Tdp2 was associated with a decrease in HBV CCC DNA. (A) and (B). HEK293 cells in 6 cm dishes were cotransfected with the envelope-defective HBV (5 μg) and Tdp2 (+) or empty vector (-) (5 μg) plasmid. Five days post-transfection the cells were harvested and analyzed for protein expression and viral DNA levels. (A) HBV core DNA was extracted and analyzed by Southern blotting (top panel). Tdp2 and HBc levels were measure by western blotting (2nd and 3rd panels). β**-**actin was measured by western blotting as a loaded control (4^th^ panel). (B) HBV PF DNA were extracted and analyzed by Southern blotting. PF DNA samples were Dpn I-digested to degrade plasmid DNA (lanes 1–4). Plasmid fragments at the bottom of the agarose gel are indicated. To further verify the identity of CCC DNA, the Dpn I digests were boiled to reduce RC and double-stranded linear (DSL) DNA species (as well as plasmid fragments) to single strands whereas the supercoiled CCC DNA remained circular (lanes 5–8), or boiled and then digested to linearize the CCC DNA (lanes 9–12). PF DNA extracted from HepAD38 cells was loaded in lane 13 as a positive control for the mobility of the RC, DSL, and CCC DNA species. (C) Quantitative analysis of HBV core and PF DNA. The triangles at the bottom represent the graded amounts of HBV and Tdp2 (+) or empty vector (-) plasmid: left bar, 7.5 μg HBV plasmid and 2.5 μg Tdp2 (+) or empty vector (-) plasmid; middle bar, 5 μg each HBV and Tdp2 (+) or empty vector (-) plasmid; right bar, 2.5 μg HBV plasmid and 7.5 μg Tdp2 (+) or empty vector (-) plasmid. The indicated viral DNA ratios were normalized to the corresponding DNA ratios from empty vector transfection (set to 100%) and are reported as the mean relative ratios ± standard error of the mean of three independent experiments. The asterisks (*) indicate a significant difference in CCC/core RC at HBV:Tdp2 plasmid transfection ratios of 5 μg: 5μg and 2.5 μg: 7.5 μg, compared to corresponding HBV:empty vector transfections (P < 0.05; unpaired, two-tailed t-test). CCC, CCC DNA; DSL, DSL DNA; RC, RC DNA.

To further examine the effect of Tdp2 on CCC DNA formation, we overexpressed Tdp2 in HBV replicating cells. Efforts to overexpress Tdp2 in HepG2 cells were unsuccessful due to the relatively low efficiency of transfection. Since HEK293 cells are transfected at high efficiency and can support HBV CCC DNA formation [11], they were cotransfected with varying ratios of pCMV-HBV/ENV^-^ and Tdp2 expressing plasmid. The HBV replication plasmid defective in viral envelope protein expression (HBV/ENV^-^) was chosen for these experiments since the lack of envelope proteins leads to enhanced intracellular amplification of CCC DNA and allowed better detection and quantification of HBV CCC DNA by Southern blot analysis in these cells [11, 19]. Western blot analyses verified Tdp2 overexpression (Fig 4A and 4C, bottom). HBV CCC DNA, normalized to core RC DNA, was modestly but reproducibly decreased with increasing Tdp2 expression, with greater inhibition of CCC DNA formation being observed with cotransfection of a higher amount of Tdp2-expressing DNA (Fig 4B and 4C, top). PF-RC DNA levels, normalized to core RC DNA, were not significantly affected at any ratio tested (Fig 4C, bottom). Thus, these results demonstrated that Tdp2, when overexpressed, could indeed modestly suppress HBV CCC DNA formation.

### Tdp2 knockout modestly reduced DHBV CCC DNA formation

**Fig 5:**
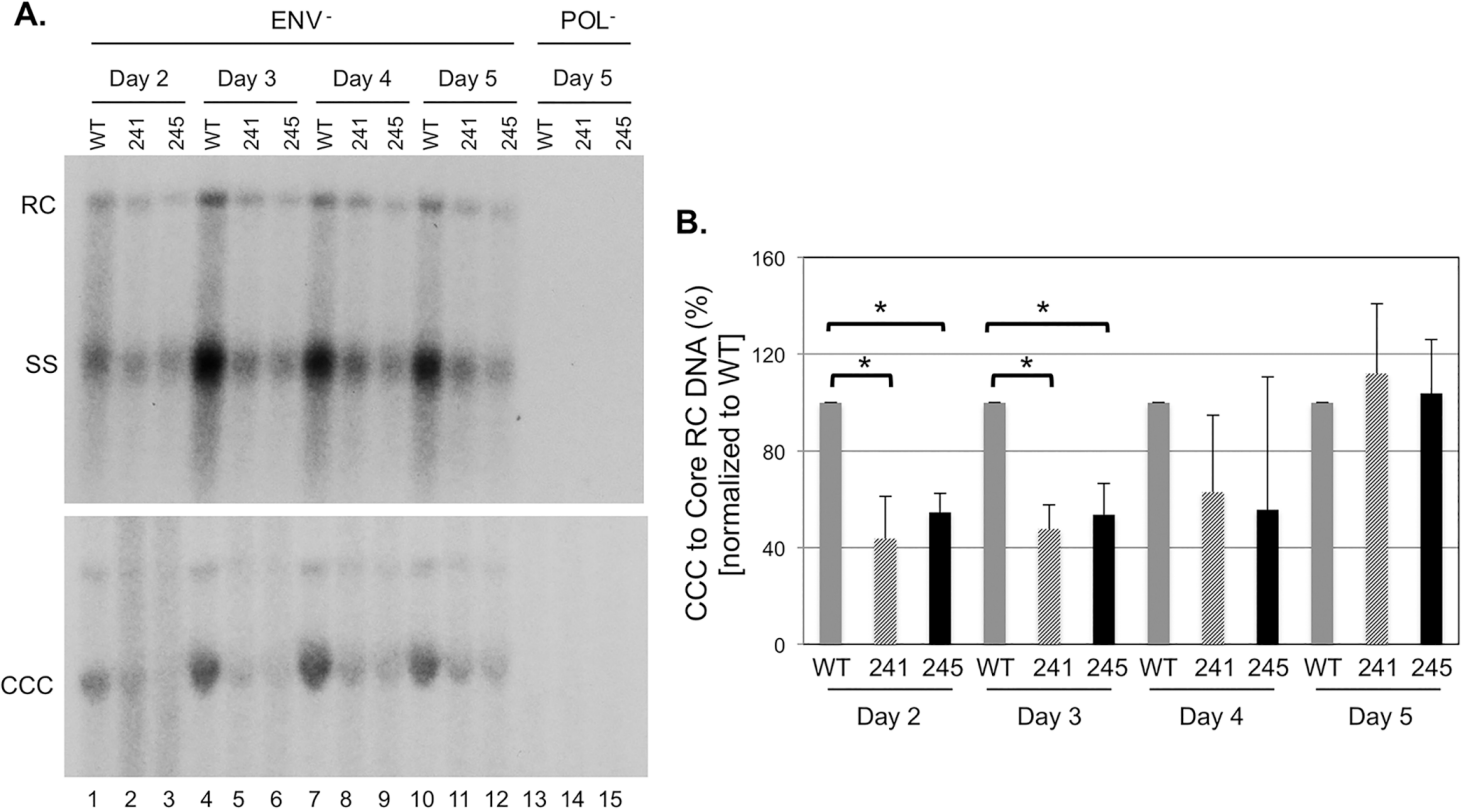
Tdp2 knockout modestly suppressed DHBV CCC DNA formation in HepG2 cells. HepG2 cells, with (241, lanes 2, 5, 8, 11, 14; 245, lanes 3, 6, 9, 12, 15) or without (WT, lanes 1, 4, 7, 10, 13) Tdp2 knockout, were transfected with the envelope-defective DHBV replication plasmid (lanes 1–12), or the polymerase-defective DHBV plasmid as a negative control (lanes 13–15). On the indicated days post-transfection the cells were harvested and analyzed for protein expression and viral DNA levels. A. DHBV core (top) and PF DNA (bottom) levels analyzed by Southern blot analysis. B. Levels of DHBV CCC DNA on the indicated days post-transfection were normalized to those of the core RC DNA and the ratios of CCC/core RC DNA in the Tdp2 KO cells were compared to those in the WT cells, which were set at 100. The asterisks (*) in B indicate statistically significant differences (P < 0.05; unpaired, two-tailed t-test). CCC, CCC DNA; RC, RC DNA; SS, single-stranded DNA [full-length (-) strand].

It was recently reported that RNAi-mediated Tdp2 knockdown modestly decreased DHBV CCC DNA levels in human hepatoma cells at early time points during a DNA transfection experiment [25]. To determine the effect of Tdp2 knockout on DHBV CCC DNA formation, we transfected DHBV replication plasmid to Tdp2-knockout HepG2 cells and measured DHBV CCC DNA formation over a time course from day 2 to day 5 post-transfection. As with the HBV transfection, above, a DHBV replication plasmid defective in viral envelope protein expression was chosen for these experiments since the lack of envelope proteins leads to increased intracellular amplification of CCC DNA and allowed easy detection and quantification of DHBV CCC DNA [17]. DHBV core DNA was lower in the Tdp2 knockout cells (Fig 5A, top) due to the decreased levels of DHBV core protein (not shown) as in the case of HBV above. When normalized to the core RC DNA, DHBV CCC DNA was modestly decreased (by ca. 2-fold) in the Tdp2 knockout HepG2 cells at days 2 and 3 post-transfection (Fig 5). By days 4 and 5 post-transfection, the DHBV CCC DNA in the knockout cells reached similar levels as those in the parental HepG2 cells.

## Discussion

HBV CCC DNA formation must be preceded by removal of RT, covalently linked to the genomic RC DNA. Mechanistically, we can envision two possibilities: RT is removed directly by cleavage of the phosphotyrosyl bond or indirectly by an endonuclease cleaving near the 5’ end of the (-) stand DNA. In this study, we have investigated the first possibility because the phosphotyrosyl bond between RT and the 5’ end of the (-) strand of RC DNA is identical to the tyrosyl-5’ DNA linkage of cellular Topo II-DNA adducts that are efficiently repaired by Tdp2 [20]. Consistent with the model, we have demonstrated that Tdp2 can specifically cleave the phosphotyrosyl linkage between RT and RC DNA *in vitro*, independent of DNA or protein size but strictly dependent on the Y residue in RT that is attached to DNA. A recent report also suggested that native RC DNA attached to presumably full-length RT was also susceptible to Tdp2 cleavage [25]. These results are in contrast to the cleavage of Topo II-DNA linkage by Tdp2, which apparently requires the proteolysis of the Topo II protein [32] possibly via the proteosome *in vivo* [33, 34]. However, it remains possible that under certain circumstances, RT linked to RC DNA is proteolytically digested first into a truncated form before Tdp2 mediated release of the remaining RT peptide from RC DNA, or via some other mechanism(s) (see below).

Recent crystal structure studies on Tdp2 suggest that single stranded DNA at least 2–3 nt long covalently attached to a tyrosine residue may be needed for Tdp2 recognition [35, 36]. However, we have found that Tdp2 is able to remove a single dNMP (any one of the 4 natural nucleotides), short DNA oligomers several nt long, as well as long DNA strands (over 100 nt in length), attached to the RT protein (this report and [9, 24]), suggesting precise recognition of the Tyr residue in the active site, without the need for any extensive DNA interactions. The discrimination of Tdp2 against the phosphoseryl or phosphothreonyl linkages at the RT protein-DNA junction further supports strict recognition of the tyrosyl residue at the protein-DNA junction by the Tdp2 active site.

As mentioned above, deproteination of RC DNA may be accomplished through another mechanism that does not require Tdp2. Interestingly, when we knocked down Tdp2 expression in HepG2 cells replicating HBV, CCC DNA was modestly increased, suggesting that in HepG2 cells Tdp2 may function to block, rather than facilitate, CCC DNA formation via the intracellular amplification pathway. Furthermore, HepG2-NTCP cells remained susceptible to HBV infection, indicating that Tdp2 was dispensable for CCC DNA formation, favoring a model where RT is removed indirectly through cleavage by a still elusive endonuclease near the 5’ end of the (-) strand DNA. Such a model is also supported by genetic data revealing that the first nucleotide attached to the (-) strand DNA is lost during CCC DNA formation [37]. We also found that overexpression of Tdp2 led to a decrease in HBV CCC DNA levels in HEK293 cells, suggesting that Tdp2 mediated removal of RT from RC DNA might actually be counterproductive for HBV CCC DNA formation. However, it is important to recall, that Tdp2 also plays a role in signal transduction, and hence, small differences in CCC DNA production in cells lacking or overexpressing Tdp2 might be caused by changes in the physiological state of these cells. In light of this possibility, the small decrease in DHBV CCC DNA formation we observed here in Tdp2-knockout HepG2 cells and that observed by others when Tdp2 expression was inhibited by RNA interference [25], both only at the earlier time points of the transiently transfected cells, should be interpreted with caution. If Tdp2 indeed plays a positive role in DHBV CCC DNA formation during the early time points of viral replication during transient transfections, its role can apparently be substituted by other factors later in those experiments, as suggested earlier [25]. It is notable, however, that differences exist in RC DNA deproteination and CCC DNA formation between DHBV and HBV. In general, DHBV CCC DNA formation is much more efficient than HBV in HepG2 and other cell lines [11, 38]. Furthermore, DHBV CCC DNA, not PF-RC DNA, is the predominant species found in cell cultures as well as in the liver, in contrast to HBV that accumulates much more PF-RC DNA than CCC DNA in cell cultures [11, 38]. However, both our study here and the previous report tested only DHBV CCC DNA formation in the context of intracellular amplification in heterologous human hepatoma cells.

**Fig 6:**
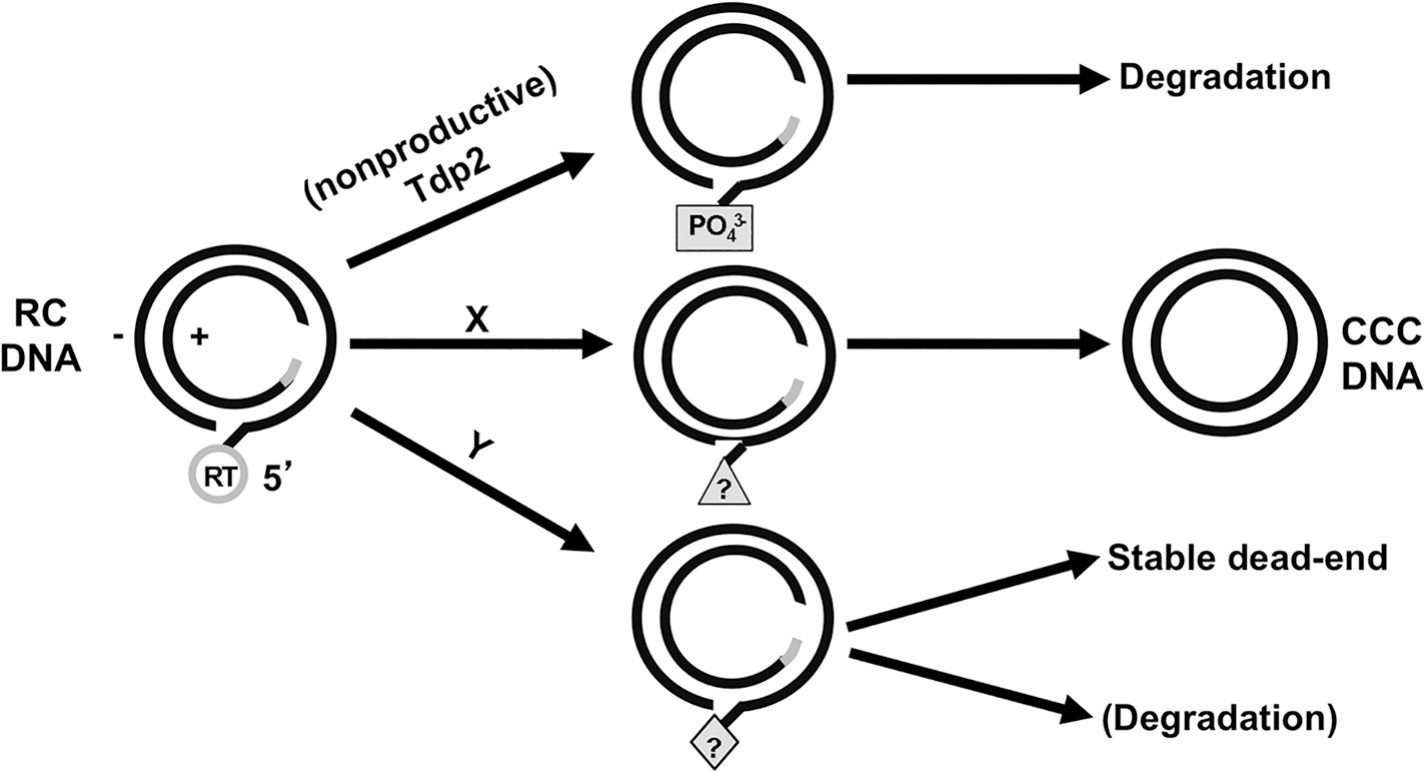
Tdp2 may direct HBV RC DNA down a pathway of deproteination nonproductive for CCC DNA formation. Tdp2 cleavage of the phosphotyrosyl linkage between the viral RT and RC DNA generates a free 5’- phosphate (PO_4_^3-^) leaving the 5’-end of the (-) strand susceptible to cellular nuclease degradation. X and Y represent two other alternative pathways of RC DNA deproteination. Pathway X depicts productive CCC DNA formation through an undefined PF-RC DNA intermediate(s). Pathway Y represents additional pathway(s) that could produce PF-RC DNA species that could accumulate and have been detected under certain conditions. The triangle and diamond with question marks represent unknown (-) strand 5’ end structures.

Significantly, HBV PF-RC DNA levels were left unchanged by Tdp2 knockdown or overexpression, suggesting that Tdp2-derived PF-RC DNA may not accumulate in these cells. The fact that stable PF-RC DNA does accumulate in HBV replicating cells in culture [11, 14, 16] and the mouse liver [15] indicates the existence of one or more additional RC-DNA deproteination pathways that can lead to the formation of a stable PF-RC DNA product. Based on the findings presented here and from previous studies [11], we propose the following model (Fig 6) to explain how Tdp2 suppresses CCC DNA formation in tissue culture cells. When Tdp2 is present it can deproteinate RC DNA, generating a PF-RC DNA species that is incompetent for CCC DNA formation but is now more susceptible to degradation by cellular nucleases and fails to accumulate. Conversely, a lack of, or decrease in, Tdp2 promotes RT removal via alternative mechanisms with at least one being compatible with CCC DNA formation.

## Materials and Methods

### Plasmids

pCMV-HBV/ENV^-^ and pCMV-HBV/POL^-^ direct the expression of the HBV pgRNA under the cytomegalovirus immediate early promoter but are defective in the expression of HBV surface proteins and polymerase protein, respectively [11]. Similarly, pCMV-DHBV/ENV^-^ and pCMV-DHBV/POL^-^ direct the expression of the DHBV pgRNA under the cytomegalovirus immediate early promoter but are defective in the expression of DHBV surface proteins and polymerase protein have been described [11]. pcDNA3.1-Tdp2 expresses the full-length human Tdp2 protein [20].

### Cells

HepAD38 cells, derivatives of the human hepatoma cell line HepG2 that express HBV pgRNA under the control of a tetracycline-repressible promoter [31], were maintained in Dulbecco modified Eagle medium-F-12 medium supplemented with 10% fetal bovine serum (complete DMEM-F12) and 5 μg/ml of tetracycline until induction. The human embryonic kidney cell line HEK293 was cultured in complete DMEM-F12.

### CRISPR-mediated Tdp2 knockout in HepG2 cells

HepG2 cells expressing Cas9 and HepG2 cells expressing Cas9 and NTCP have been described previously [30]. The following two sgRNAs were selected for targeting Tdp2: 5’ GAGCACCAGAGGGCATGCT 3’ (cell line 221) and 5’ GGCCGAGAACGACTGGGAGA3’ (cell lines 241–245). The sequences were derived from Table 8 in reference [39]. sgRNAs were cloned into lentivirus vectors as described in [30]. Lentivirus vectors were produced in 293T cells and used for infection of HepG2/NTPC/Cas9 cells as described previously [30]. Cell lines were established from clones derived from the original pool of lentivirus infected cells.

### Transient transfection

HepG2 cells were transfected using Fugene 6 (Roche) as previously described [40] or using lipofectamine 3000 (Invitrogen) per manufacture’s instruction. HEK293 cells were transfected using the CalPhos Mammalian Transfection kit (Clontech) [40]. Cells were harvested on day 5 post-transfection unless otherwise indicated.

### Virus and infections

HBV was concentrated one hundred-fold from the culture medium of HepAD38 cells with 6% polyethylene glycol (PEG) and resuspended in serum-free DMEM/F12 medium. Infection of HepG2 cells was carried out in the presence of 4% PEG in complete serum-free medium (DMEM/F12, pyruvate, non-essential amino acids, and penicillin/streptomycin) containing 2% DMSO with an estimated 50 genome equivalents per cell. Medium was replaced with complete medium containing 10% fetal calf serum and 2% DMSO 24 hours after infection. Cultures were incubated for 5–10 days.

### Immunofluorescence

For immunostaining, cells were fixed in 96-well plates with 4% paraformaldehyde for 10 minutes and processed for immunofluorescence with HBc monoclonal antibody C1-5 (Santa Cruz Biotech). The fraction of HBc positive cells was determined with an ImageXpress Micro automated microscope (Molecular Devices). Images from 16 preset positions at 10x magnification with two channels (DAPI, Cy5) were collected from each 96 well. Images were analyzed with MetaXpress imaging and analysis software using the multi-wavelength cell scoring module.

### siRNA

ON-TARGETplus SMARTpool small interfering RNA (siRNA) against human Tdp2 was purchased from Dharmacon RNA Technologies (L-017578-00). Four days after HBV replication was induced by removal of tetracycline from the culture medium, HepAD38 cells were transfected with siRNA at a final concentration of 100 nM using Dharmafect 1 Transfection Reagent (Dharmacon RNA Technologies) according to the manufacturer's specifications for HepG2 cells. An ON-TARGETplus Non-targeting siRNA was also purchased from Dharmacon RNA Technologies (D-001810-01) as a negative control. The cells were harvested 3 and 5 days post-transfection for DNA and protein analysis.

### DNA and protein analysis by Southern and western blotting

HBV core and PF DNAs were extracted as previously described and analyzed by Southern blotting [11]. Whole-cell extracts from a portion of transfected cells were fractionated by sodium dodecyl sulfate-polyacrylamide gel electrophoresis (SDS-PAGE) and analyzed by western blotting for Tdp2 using polyclonal rabbit anti-human Tdp2 [20] or a commercial anti-Tdp2 antibody (Bethyl Labs) and HBV core protein using a mouse monoclonal antibody specific for the N-terminal end of core protein [41] respectively. To control for loading, western blotting for flap endonuclease 1 (FEN-1) or β**-**actin was carried out using monoclonal mouse anti-FEN-1 (BD Transduction Laboratories) or β**-**actin (Cell Signaling Technology). Southern and western blotting data were quantified by phosphorimaging and densitometry.

### Tdp2 cleavage assays

Full-length human Tdp2 recombinant protein with glutathione *S*-transferase (GST) tag was purchased from Abnova (H00051567-P01). HBV core DNA (600 pg RC DNA equivalent) extracted from cytoplasmic nucleocapsids with proteinase K digestion was incubated with recombinant Tdp2 (final concentration, 320 nM) at 37°C for 1 hr, in 1x Tdp2 buffer (25 mM Tris-HCl, pH 8.0, 130 mM KCl, 1 mM dithiothreitol (DTT), 10 mM MgCl_2_) containing 1X Ethylenediaminetetraacetic acid (EDTA)-free protease inhibitor cocktail (P.I.s, Roche). The reaction products were phenol/chloroform extracted and ethanol precipitated using pellet paint (Novagen) plus tRNA. The purified DNA was then digested with the Sfc I restriction endonuclease (NEB) for 2 h at 25°C followed by labeling with [α-^32^P]-TTP for 2 h at 25°C using Klenow (NEB). RNase digestion was used to remove the RNA primer attached to the 5’ end of the plus strand DNA. The digested DNA was extracted as above by phenol/chloroform extraction and pellet paint precipitation. Pellets were resuspended in 2X formamide loading buffer (Ambion) and boiled for 5 min prior to resolve by urea PAGE. All reaction products were visualized by autoradiography.

DHBV protein priming assays were carried out as previously described [26–28]. Briefly, the following components were added to a 10 μl reaction mixture: GST-MiniRT2, epsilon (ε) RNA, [α-^32^P]-dGTP and the other three unlabeled dNTPs, and TMnNK buffer (10 mM Tris-HCl, pH 8.0, 1 mM MnCl_2_, 15 mM NaCl, 20 mM KCl). For *trans*-complementation priming assays [26], purified N-terminal hexahistidine tagged (6xHis)-TP or its mutant derivative His-TP/Y96F, and GST-RT were added to the priming reaction mixture instead of the MiniRT2. Protein priming reactions were conducted at 30°C for 2 h. Tdp2 cleavage reactions using *in vitro* protein priming products were carried out as previously described [9, 24]. SDS-PAGE was used to visualize the RT protein attached to radiolabeled DNA, and urea-PAGE for detection of DNA oligomers released from RT following Tdp2 cleavage.
